# Transvaginal small bowel evisceration: Case report and review of literature

**DOI:** 10.1016/j.ijscr.2022.107322

**Published:** 2022-06-24

**Authors:** Raef Alfraidi, Nourah Abdulaaly, Ashwag Alharbi, Helayal Almodhaiberi, Bander Ali, Hassan Sabagh

**Affiliations:** a Department of General Surgery, Prince Sultan Military Medical City, Riyadh, Saudi Arabia; b Department of Obstetrics and Gynaecology, Prince Sultan Military Medical City, Riyadh, Saudi Arabia

**Keywords:** Evisceration, Transvaginal, Small bowel, Hysterectomy, Case report

## Abstract

**Introduction:**

Transvaginal evisceration is a rare life-threatening condition that is usually seen in postmenopausal women with past history of gynaecological surgery.

**Presentation of case:**

A 54-year-old woman presented with sudden-onset abdominal pain and protrusion of a mass through the vagina after her grandson jumped on her abdomen while at play. She had undergone laparoscopic total hysterectomy and bilateral salpingo-oophorectomy 9 months earlier. Physical examination revealed an intestinal loop in the vagina. She was immediately taken to the operating room. Laparotomy was performed and the prolapsed intestine was reduced. The bowel from the duodenojejunal junction to the ileocecal valve looked healthy, with no areas of ischemia. The rupture in the vaginal vault was repaired with Vicryl. The postoperative course was uneventful, and the patient was discharged on day 5 after the surgery.

**Discussion:**

Less than 100 cases of transvaginal evisceration have been reported. The condition, which is more common in postmenopausal women, usually presents with sudden-onset abdominal pain. Some patients may be asymptomatic and only complain of swelling at the introitus. Diagnosis can usually be made by visual examination. Immediate surgery is necessary to reduce the risk of intestinal ischemia and necrosis.

**Conclusion:**

Transvaginal evisceration must be suspected in any woman presenting with sudden-onset abdominal/pelvic pain. Awareness of this rare condition is necessary to minimize mortality.

## Highlights

• Transvaginal evisceration is a rare life-threatening condition.
• It most commonly affects postmenopausal women with past history of hysterectomy.
• Diagnosis can usually be made by physical examination alone.
• Immediate surgical intervention is necessary to reduce risk of intestinal ischemia.

## 1 Introduction

Transvaginal evisceration of bowel is extremely rare. Prompt recognition and surgical management are crucial. We report a postmenopausal woman who presented with transvaginal evisceration and was successfully treated with abdominal surgery. This work has been reported in line with the SCARE criteria [1].

## 2 Presentation of case

In March 2022, a 54-year-old woman presented to the emergency department complaining of lower abdominal pain that had started suddenly about 10 h earlier after her grandchild playfully jumped on her abdomen. The pain was localised to the lower abdomen, and had slowly increased in severity over time. There were no relieving or aggravating factors. While passing stools before her presentation she had noticed an intestinal loop protruding through the vagina and so came immediately to the emergency room. On examination, she was conscious, alert, oriented, and comfortable lying on the examination couch. Her vital signs were stable. The abdomen was soft and lax, with only mild lower abdominal tenderness. The bowel sounds were normal. Vaginal examination revealed a bowel loop protruding through vagina. The bowel was pink in color, with no areas of discoloration or signs of ischemia. There was no blood or vaginal discharge. The patient's past history was significant for laparoscopic total hysterectomy and bilateral salpingo-oophorectomy performed 9 months earlier for uterine fibroid.

**Fig. 1:**
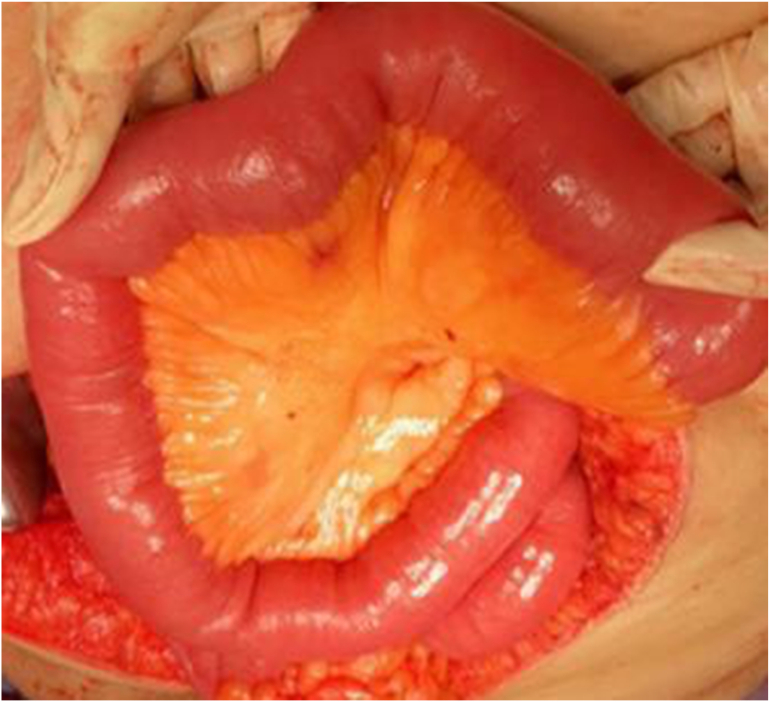
Intra-operative finding showed healthy bowel, with no signs of ischemia or any other injury.

Venous blood was drawn and sent to the laboratory; it showed the following: white blood cell count, 8.8 × 10^9^/L; hemoglobin, 13 g/dL; platelet count, 240 × 10^9^/L; and blood lactate1.1 mmol/L. Due to the obvious findings on physical examination, imaging was not considered necessary, and the patient was taken directly to the operating room, where we were joined by doctors from obstetrics and gynaecology. Laparotomy was performed with a lower abdominal transverse incision. The bowel, from the duodenojejunal junction to the ileocecal valve, looked healthy, with no signs of ischemia or any other injury (Fig. 1). There was an opening between the edges of the vaginal vault, which was closed by the obstetrics and gynaecology surgeons using Vicryl®1 sutures, while the bowel kept away from the vault. A drain was inserted, and the abdomen was closed. The patient's postoperative course was uneventful. The drain was removed and the patient discharged 5 days after the surgery with advice to attend follow-up in the obstetrics and gynaecology outpatient department.

## 3 Discussion

Transvaginal evisceration was first described by Hyernaux as far back as 1864; since then less than 100 cases have been reported [2], [3]. Most patients (75 %) had history of previous gynaecological surgery such as repair of vaginal prolapse or hysterectomy. Vaginal evisceration without past history of gynaecological surgery is extremely unusual [4], [5], [6].

Transvaginal evisceration is more common in postmenopausal women. The risk factors include postoperative infection, hematoma formation, smoking, long-term steroid treatment, diabetes mellitus, malnutrition, radiation therapy, chronic constipation, vaginal vault prolapse, cervical cancer, and severe cuff atrophy [4], [6], [7], [8]. In premenopausal women, the risk factors include obstetric injury, vaginal cuff infections, vaginal trauma, and unusual sexual practices; sexual intercourse alone has also been identified an important precipitating factor in this age-group [9], [10].

Vaginal cuff dehiscence (VCD) may occur in 0.032 % of patients after abdominal or vaginal hysterectomy; the risk is reported to be three-fold higher after laparoscopic hysterectomy [8], [11]. The site of the VCD is usually the posterior fornix of the vagina, where the intra-abdominal pressure is highest [8], [12], [13]. Following VCD, there is a 67 % probability of evisceration of intraperitoneal content [14].

The patient usually presents with sudden-onset abdominal or pelvic pain, with or without vaginal bleeding and protrusion of a mass through the vagina; however, some patients may only complain of an undefined swelling at the vaginal introitus [15]. Transvaginal evisceration has been reported from 5 days to 30 years after surgery [16], [17].

Most commonly, it is the terminal ileum that prolapses; this is because of its long mesentery and mobility and its location. The omentum, fallopian tubes, appendix, colon, and appendices epiploicae may also be involved [6].

The mortality rate in patients with transvaginal evisceration can be as high as 10 %, the risk being especially high in those with strangulated bowel [18]. Therefore, rapid diagnosis and treatment are crucial. Diagnosis is usually made on direct visualization during pelvic examination. Imaging examinations are not necessary; however, if time permits, a contrast-enhanced CT scan of the abdomen and pelvis could sometimes be helpful for the surgeon [19].

Surgery is usually performed using a wide laparotomy; this allows the surgeon to perform a thorough intra-abdominal inspection and meticulous peritoneal lavage. However, transvaginal, combined vaginal–abdominal, and laparoscopic–vaginal approaches have all been successfully used [15]. The transvaginal approach is suitable for patients with easily reduced viable bowel and no sign of peritonitis [20]; however, this approach should be avoided if there is a high vaginal defect or strangulated bowel. The combined vaginal–abdominal approach is indicated in patients with an incarcerated but viable bowel [6]. Some authors have reported good results with laparoscopic repair [6], [20]. It should be noted that, even with surgical intervention, the mortality rate can be as high as 6 % [18].

Kowalski et al. have proposed some surgical steps for prevention of this serious complication; 1) restoration of normal vaginal axis; 2) anastomosis of the stumps of the supporting ligaments of the pelvis to the angles of the vagina; 3) preservation of vaginal length, and 4) maintenance of vaginal integrity with application of oestrogen if necessary [4].

## 4 Conclusion

Complaints of pelvic/abdominal pain or watery discharge in a patient with history of previous gynaecological surgery should arouse suspicion of transvaginal evisceration. It is a serious life-threatening condition requiring immediate surgical intervention.

## Patient perspective

Patient was satisfied with her treatment and she was given regular follow up.

## Consent

Written informed consent was obtained from the patient for publication of this case report. A copy of the written consent is available for review by the Editor-in-Chief of this journal.

## Sources of funding

This research did not receive any specific grant from funding agencies in the public, commercial, or not-for-profit sectors.

## Ethical approval

In our institute, ethical approval is exempted, depend on acquired patient consent.

## Guarantor

The Guarantor is DR. Ashwag Alharbi.

## Registration of research studies

We don't need to register this work.

## CRediT authorship contribution statement

All authors contributed to manuscript preparation, manuscript editing, manuscript review.

## Declaration of competing interest

The authors have no conflict of interest.
